# Creating a Public Health Community of Practice to Support American Indian and Alaska Native Communities in Addressing Chronic Disease

**DOI:** 10.5888/pcd16.190193

**Published:** 2019-08-15

**Authors:** Shawna L. Williams, Alexis Kaigler, Amy Armistad, David K. Espey, Bruce B. Struminger

**Affiliations:** 1 Centers for Disease Control and Prevention, National Center for Chronic Disease Prevention and Health Promotion, Division for Heart Disease and Stroke Prevention, Atlanta, Georgia; 2 ECHO Institute, University of New Mexico Health Sciences Center, Albuquerque, New Mexico; 3 Centers for Disease Control and Prevention, National Center for Chronic Disease Prevention and Health Promotion, Atlanta, Georgia

Across the lifespan, American Indian and Alaska Native (AI/AN) people have higher rates of chronic disease, injury, and premature death than some racial/ethnic groups in the United States (1,2). For example, AI/AN adults have a higher prevalence of obesity, are twice as likely to have diabetes, and are more likely to be current smokers than their non-Hispanic white counterparts (3). Rates of death due to stroke and heart disease are also higher among AI/ANs than among members of some racial and ethnic groups (4,5).

Recognizing AI/AN communities have their own cultural strategies for chronic disease prevention and control, the Centers for Disease Control and Prevention (CDC) created the Good Health and Wellness in Indian Country (GHWIC) program to integrate the knowledge those communities possess into a coordinated approach to healthy living and chronic disease prevention. The program also sought to reinforce efforts in Indian Country to advance policy, systems, and environmental (PSE) improvements to make healthy choices easier for all community members.

CDC launched GHWIC in 2014 as a 5-year, $78-million initiative and funded 23 recipients (Figure). To prevent and control diabetes, cardiovascular disease, and other chronic diseases, 12 tribes (Component 1 recipients) implemented community-selected strategies to reduce commercial tobacco use and exposure, improve nutrition and physical activity, and link community programs to clinical services. GHWIC also supported 11 tribal organizations (Component 2 recipients) to provide leadership, technical assistance, and resources to AI/AN tribes in each administrative area of the Indian Health Service. Eleven tribal epidemiology centers and the Urban Indian Health Institute supported GHWIC’s surveillance and evaluation activities (6).

**Figure:**
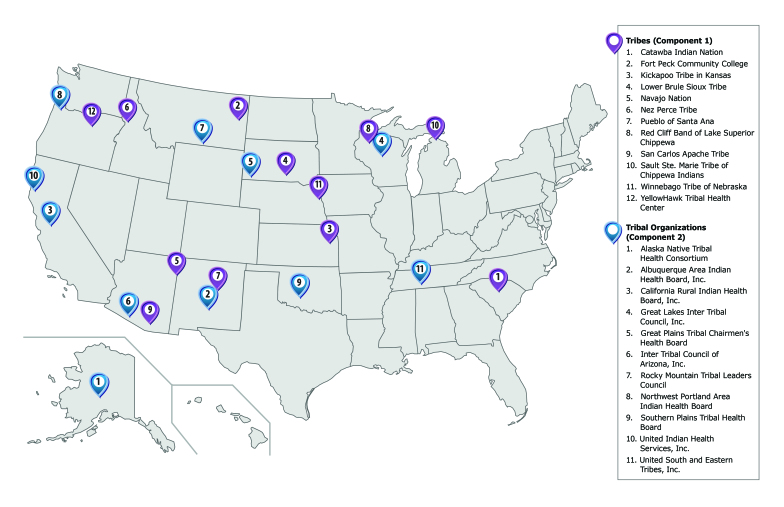
The 23 recipients of the Good Health and Wellness in Indian Country program, funded in 2014 by the Centers for Disease Control and Prevention.

## Using the ECHO Model for Public Health Practice

The ECHO model (Extension for Community Healthcare Outcomes) was created at the University of New Mexico in 2003 to build provider capacity in hepatitis C treatment in underserved communities (7). ECHO combines evidence-based education, workforce development, and collaborative problem solving to increase practitioners’ capacity in specialty areas (5). The model uses videoconferencing and subject matter expertise to facilitate case-based learning among practitioners and to share best practices (5). During a typical ECHO session, after a brief didactic lecture, providers from multiple clinical sites present their patient cases to a multidisciplinary team of mentors for discussion, feedback, and collaborative problem solving and patient health management (5).

GHWIC was the first program to adopt the ECHO model for public health practice to increase public health practitioners’ capacity in evidence-based chronic disease interventions by building and supporting a virtual community of practice among GHWIC recipients. A community of practice refers to a group of people engaged in collective learning and problem solving in a particular area of interest or work. According to Wenger and Trayner, a community of practice has 3 key characteristics: the topic or domain; the group or community of learners; and the practices that emerge from the community of practice (8). For GHWIC, the domain was chronic disease prevention in AI/AN communities; the group comprised GHWIC tribal public health workers, CDC staff, and other partners; and the practice captured stories, successes, and lessons learned.

CDC partnered with Project ECHO in the first year of GHWIC, and in collaboration with the Urban Indian Health Institute, CDC launched 2 types of GHWIC–ECHO sessions to support program objectives. The first type was a monthly videoconference held separately for Component 1 and Component 2 recipients, which included representatives from CDC and the Urban Indian Health Institute. Each session was co-led by a CDC facilitator and a Project ECHO liaison. Early sessions included a didactic presentation on an intervention or resource, such as a CDC framework for a community health needs assessment given by CDC or an invited partner organization, followed by 2 or 3 recipients who shared their experiences on the topic. The facilitator invited recipients in advance to share their experiences during the session to encourage discussion. The second type was the quarterly “All Hands” GHWIC–ECHO session, which included all recipients. CDC invited external organizations to provide a brief presentation about resources and partnerships of interest to GHWIC recipients in the All Hands GHWIC-ECHO sessions. For instance, 1 session included a presentation by a member of the Southcentral Foundation, who gave an overview of the Nuka System of Care, an innovative customer-owned model for providing health care in Alaska. CDC invited subject matter experts to join both types of sessions as needed. For example, for a discussion of challenges in community buy-in of a tobacco cessation program, a tobacco cessation expert from CDC and a tribal community were invited to describe resources and possible solutions.

## Evaluating the GHWIC–ECHO Sessions

Recipients who participated in the GHWIC–ECHO sessions self-reported that using the videoconferencing platform helped them build relationships with their counterparts and that seeing each other on camera strengthened connections that were beneficial both on screen and when they met in person. Participants indicated that the platform’s chat feature was useful and that the sessions were used as a means to start conversations and exchange resources both publicly and privately. AI/ANs have cultural communication customs that include not interrupting elders who are speaking. One tribal member commented that the chat feature accommodated this cultural custom by allowing tribal participants to ask questions without interrupting the speaker during a GHWIC-ECHO session. The videoconference platform also allowed participants to simultaneously view documents, slides, and other media on a shared screen.

In addition, a GHWIC–ECHO Workgroup, comprising the facilitator, GHWIC recipients, and staff members of CDC, Project ECHO, and the Urban Indian Health Institute convened monthly to review the previous GHWIC–ECHO session and discuss evaluation results, priority topics, and ideas for increasing engagement in GHWIC–ECHO sessions.

From January 2015 through June 2015, post-session evaluations were collected via an online survey that included 4 questions on a Likert scale about the quality and value of the GHWIC–ECHO sessions. The evaluation response rate averaged 18% during the 6-month period.

## Evaluation Results

Of the 126 responses to the online survey, 55% of respondents rated the overall quality and value of the GHWIC–ECHO sessions as either excellent or very good, 36% rated sessions good, and 9% rated them fair.

Early evaluation results indicated a need to revamp the sessions to give recipients more opportunities to actively participate and that the sessions needed to better incorporate the unique knowledge, perspectives, and experiences of tribal participants. Overall, the evaluation indicated that the initial sessions over-emphasized presentations by CDC staff members and did not fully reflect how tribes share and communicate.

In response to recipients’ feedback, CDC modified the GHWIC–ECHO session format to promote greater engagement and a more conducive environment for recipients to exchange knowledge and solutions with each other. Significant changes included topic selections based on priorities and interests expressed by recipients through the post-session evaluations; shifting from CDC to recipient presentations as the primary focus of each session; recruiting an American Indian session facilitator with GHWIC and public health expertise; and circulating discussion questions to recipients before the sessions to encourage more robust discussions. The facilitator also used a web-based polling application as a tool to increase engagement and interaction among participants by inviting them to respond to questions via the web or text message. Poll questions included “ice-breakers” (eg, “Who do you think will win the Super Bowl?”) and questions related to program activities (eg, “Who should be a part of team-based care?”).

The newly formatted monthly sessions included a recipient presentation of his or her GHWIC project as a case. Examples of cases included a presentation on community health assessments, a plan for a tribal food sovereignty coalition, and ideas for building healthy communities through PSE change strategies. The quarterly All Hands sessions continued to feature external partners for presentations of interest to the GHWIC recipients. However, presentations were selected on the basis of recipients’ topic interests, and session facilitators briefed partners in advance on how to correlate their presentation to GHWIC. For example, a representative of the National Park Service described how the Rivers, Trails, and Conservation Assistance program applied to GHWIC’s work to promote physical activity.

## Culturally Tailoring the ECHO Model for Public Health Practice

After the GHWIC–ECHO session format was revised, the post-session evaluation responses to the question “What did you like most about the ECHO session?” included the following: “The interaction and problem solving as well as shared video presentation.” “The focus on what tribes are doing to implement GHWIC. These monthly calls have gotten so much better and so much more applicable to our work. Thank you for the shift in focus!” “I thought ending the recording after the presentation was a significant improvement! There seemed to be much more interaction.”

From August 2017 through January 2018, evaluations were conducted online after each session to assess the quality and value of the new format. During this period, the online evaluation consisted of 6 questions on a Likert scale, and of the 339 participants, 14% responded. Of the 47 respondents, 34 (72.3%) rated the overall quality and value of the newly formatted sessions as excellent or very good, 13 (27.7%) rated the sessions as good, and none rated the sessions as fair or poor. Overall, recipient feedback, measured through online evaluations from January 2015 through January 2018, indicated a shift in recipient satisfaction with the GHWIC-ECHO session format.

## Applications of Exchanged Knowledge to GHWIC work

Recipients reported exchanging new ideas for implementing culturally tailored PSE approaches to improve health and wellness in their communities. By Year 3 of GHWIC, tribal recipients had adopted policies in more than 100 settings to promote healthy behaviors (9). GHWIC recipient Yellowhawk Tribal Health Center promoted access to healthy foods in 37 settings and frequently exchanged ideas during the GHWIC–ECHO sessions about gardening classes and ways to promote their community garden (7). The presentation on their community garden and greenhouse project as well as a food sovereignty presentation by the American Indian Health and Family Services, a sub-recipient of Great Lakes Inter-Tribal Council, Inc, resulted in the formation of a sustainable food systems workgroup comprising GHWIC recipients that met quarterly via videoconference.

The California Rural Indian Health Board, Inc, provides another example of how knowledge exchanged on GHWIC–ECHO sessions was applied to GHWIC work. They shared their “Tribal Policy, Systems, and Environmental Strategies for Preventing Chronic Disease” toolkit during a GHWIC–ECHO session that included examples of PSE strategy options for various sectors of tribal communities. As a result, the Southern Plains Tribal Health Board and the Great Lakes Inter-Tribal Council, Inc, used the California Rural Indian Health Board’s toolkit to guide their sub-recipients in shifting from individual-based interventions, such as health fairs and fun run events, to sustainable PSE interventions, like tobacco-free policies and breastfeeding initiatives. This knowledge exchange helped sub-recipients better align their activities with the objective of the GHWIC program to address chronic disease outcomes through PSE interventions.

## Lessons Learned and Future Directions

After the GHWIC-ECHO session format was adjusted, the quality and applicability of the sessions improved. Session planning and participation improved by pivoting from the use of external experts to tribal public health staff members sharing best practices and arranging for an American Indian GHWIC–ECHO session facilitator.

A unique challenge to ensuring robust recipient participation relates to the inherent structural hierarchy of CDC as the funder and tribes and tribal organizations as the recipients of funding, as well as the complex history between tribal governments and the federal government (8). Acknowledging the complex tribal–federal government history is critical to creating a safe space for tribal recipients to share their work. GHWIC–ECHO session planners strived to take this history into account and modified the GHWIC–ECHO session format to accommodate these dynamics (10).

This experience demonstrates that adopting the ECHO clinical model for public health practice is possible. Inter-peer relationship building is a critical component of the ECHO model application for public health practice, particularly in a tribal setting where trust and relationships are the foundation for sharing information. Staff members from CDC and Project ECHO also observed that when working with AI/AN partners it is important to garner their input during the planning phase to ensure that the GHWIC–ECHO sessions are culturally appropriate and useful for tribal partners.

Verbal communication, body language, eye contact, and gestures are all forms of communication, and videoconferencing enables these forms of communication in a group setting for a richer interaction than what is possible in an audio conference call. Videoconferencing facilitates communication by humanizing conversation, thus contributing to the formation of a community of practice (10).

In this novel application of the ECHO model to a public health context, success was defined by tribal partners who found value in the GHWIC–ECHO sessions and engaged in the sessions, participated in topic discussions, or connected with peers after sessions. The adapted ECHO model built a community of practice where AI/AN peers established relationships, exchanged knowledge of best practices at the community level, and shared resources with each other as well as CDC staff members. The experience of adopting the ECHO model for public health practice and culturally adapting it created a blueprint for how to initiate and support a community of practice among AI/AN peers committed to chronic disease prevention in their communities and shows promise for future expansion to other public health settings.
